# A long-headed Cambrian soft-bodied vertebrate from the American Great Basin region

**DOI:** 10.1098/rsos.240350

**Published:** 2024-07-24

**Authors:** Rudy Lerosey-Aubril, Javier Ortega-Hernández

**Affiliations:** ^1^ Department of Organismic and Evolutionary Biology and Museum of Comparative Zoology, Harvard University, Cambridge, MA 02138, USA

**Keywords:** exceptional preservation, Laurentia, Marjum Formation, Miaolingian, planktonekton, vertebrate origin

## Abstract

The fossil record suggests that chordates might have been minor components of marine ecosystems during the first major diversification of animal life in the Cambrian. Vertebrates are represented by a handful of rare soft-bodied stem-lineage taxa known from Konservat-Lagerstätten, including *Myllokunmingia* and *Yunnanozoon* from the Stage 3 of South China, and *Emmonsaspis* and *Metaspriggina* from Stage 4-Drumian deposits of northeast USA and British Columbia. Here, we describe the first soft-bodied vertebrate from the American Great Basin, a region home to a dozen Cambrian Konservat-Lagerstätten. Found in the Drumian Marjum Formation of Utah, *Nuucichthys rhynchocephalus* gen. et sp. nov. is characterized by a finless torpedo-shaped body that includes a snout-like anterior head bearing anterolateral eyes, approximately 25 thick myomeres, a large branchial chamber with a keel and approximately seven putative dorsal bars and a spiniform caudal process. Using Bayesian inference, our analysis recovers *Nuucichthys* within the vertebrate stem, closer to the crown than *Pikaia*, *Yunnanozoon* and *Myllokunmingia*, where it forms a polytomy with its Laurentian relatives, *Emmonsaspis* and *Metaspriggina*, and a scion consisting of conodonts and crown-group vertebrates. Based on the eye orientation and absence of fins*,* we tentatively reconstruct *Nuucichthys* as a pelagic organism with limited swimming abilities (planktonektic).

## 1. Introduction

The Cambrian fossil record indicates that most animal phyla had diversified and inhabited the Earth’s oceans approximately 518 million years ago [1]. The chordates—the group that includes vertebrates—were part of this early animal diversification, but they were apparently less conspicuous in marine ecosystems than they are today. The scant Cambrian fossil record of putative vertebrates includes only a handful of rare soft-bodied (i.e. non-biomineralizing) taxa (i.e. *Emmonsaspis*, *Metaspriggina*, *Myllokunmingia*/*Haikouichthys* and possibly *Yunnanozoon*) almost exclusively known from Chengjiang and the Burgess Shale [2,3], the two most fossiliferous of more than 50 Cambrian Konservat-Lagerstätten worldwide. Conway Morris & Caron [4] recently proposed to reassign fossils of *Emmonsaspis* [5,6] and specimens of uncertain affinities [7] to the genus *Metaspriggina*. In this context, *Metaspriggina* would exhibit broad palaeogeographic and stratigraphic distributions [4], ranging from the Cambrian Stage 4 to the Guzhangian of British Columbia and northeast USA. However, regarding all these fossils as congeneric might have been precipitate (J.-B. Caron 2022, personal communication), especially if it involves discarding the name *Emmonsaspis* [8]. Regardless of the precise systematic treatment, it remains that Conway Morris & Caron convincingly documented that various Cambrian strata in North America contain soft-bodied early vertebrates [4]. Surprisingly, these organisms have not been reported in the American Great Basin, a region of the western United States that has proved particularly rich in Cambrian Konservat-Lagerstätten, including the tier 2 Burgess Shale-type deposits (sensu [9]) of the Pioche, Spence, Wheeler (House Range and Drum Mountains), Marjum and Weeks formations (e.g. [10–15]). In this contribution, we fill this significant palaeogeographic gap with a new stem-group vertebrate from the Drumian Marjum Formation of Utah.

## 2. Material and methods

### 2.1. Material

The studied material consists of a single fossil (part only) discovered in the Drumian Marjum Formation in the House Range of western Utah, USA, and housed in the collections of Invertebrate Paleontology of the Natural History Museum of Utah (UMNH.IP.6084). Its precise geographic origin and stratigraphic position within the formation were not provided on the label accompanying the specimen. However, the dark grey colour of the fossil material and the lighter grey colour of the surrounding matrix are strongly reminiscent of the carbonaceous fossils found in the grey layers of the Marjum Formation exposed at a few localities in the House Range [16,17].

The Marjum Formation is part of a continuous succession of mudstone and marl interbedded with thin-bedded limestone, which were deposited in a fault-controlled basin known as the House Range Embayment. This basin developed locally within the offshore margin of a carbonate platform from the late Wuliuan to the Guzhangian [18]. It represented a deep-water quiet open-marine environment, well below wave disturbance, where conditions conducive to the preservation of organic remains repeatedly developed [19,20]. The exceptionally preserved biota from the Marjum Formation is upper Drumian in age (*Ptychagnostus punctuosus* agnostoid biozone), and inhabited the subequatorial northern margin of the palaeocontinent Laurentia [12,13,16,17].

### 2.2. Institutional abbreviations

The fossils illustrated in this contribution are housed in the collections of the Natural History Museum of Utah (UMNH.IP), Salt Lake City, USA; the Royal Ontario Museum (ROMIP), Toronto, Canada; the Smithsonian National Museum of Natural History (USNM), Washington, DC, USA; and the Yunnan Key Laboratory for Palaeobiology, Yunnan University (RCCBYU), Kunming, China.

### 2.3. Imaging

UMNH.IP.6084 was photographed dry or wet under cross-polarized illumination using a Nikon D5500 DSLR fitted with a Nikon 40 mm DX Micro-Nikkor lens and a Zeiss Axiocam 208 color camera mounted on a Zeiss Stemi 305 microscope. Interpretative drawings were created based on pictures on Photoshop CC, the software that was also used to produce the figures.

### 2.4. Phylogenetic analysis

We tested the phylogenetic position of *Nuucichthys rhynchocephalus* gen. et sp. nov. by adding this new taxon and *Emmonsaspis cambrensis* [5] (Cambrian Stage 4; Parker Slate Formation) to the matrix of Tian *et al*. [3] (electronic supplementary material, dataset S1). The list of characters remained unchanged, but the following minor changes to their coding were made: (a) characters 227 (endoskeletal fin supports) and 230 (origin of fin(s) along dorsal midline) were coded as inapplicable, rather than uncertain in *Metaspriggina*, since all types of fins are coded as absent in this taxon and (b) character 231 (distinct anal fin) was coded as absent, rather than uncertain in *Metaspriggina*, as even the best preserved *Metaspriggina* specimens do not exhibit such a fin. Composed of 313 morphological characters and 99 taxa, the new dataset was analysed through Bayesian inference in MrBayes 3.2 using the Monte Carlo Markov-chain model for discrete morphological characters [21,22] for 1 million generations (four chains), with every 1000th sample stored (resulting in 1000 samples) and 25% burn-in (resulting in 750 retained samples). Convergence was verified when effective sample size values were over 200 for all parameters and corroborated using the software Tracer v. 1.6 [23]. A similar procedure was followed after removing the vetulicolians from the dataset to test whether this exclusion would affect the phylogenetic placement of the new Marjum taxon. Vetulicolians were recovered as deuterostomes in Tian *et al*.’s analysis [3], but the position of this group within deuterostomes (e.g. [24–26]) or even its deuterostome affinities (e.g. [27–30]) remain contentious.

### 2.5. Terminology

The terminology used herein mainly follows Conway Morris & Caron [4], except for the definition of the head. We define the head as the part of the body that includes the branchial cavity and anything anterior to it, following Shu *et al*. [31,32]. The terms ‘head’, ‘head region’ or ‘cephalic region’ herein are also equivalent to the ‘cephalo-pharyngeal region’ of Holland & Chen [33]. Additionally, we use the terms ‘posterior head (region)’ and ‘anterior head (region)’ to refer to the cephalic region that includes the branchial cavity and the cephalic region anterior to it, respectively. When the branchial cavity is not well circumscribed, the ‘anterior head’ is defined as the region anterior to the anteriormost branchial structure (i.e. branchial bar/arch). The term ‘anterior head’ herein corresponds to the ‘head’ in [4,34], and the term ‘branchial’ is equivalent to the term ‘pharyngeal’ of other authors (e.g. [3]).

## 3. Results

### 3.1. Systematic palaeontology

Phylum CHORDATA [35]

Genus *Nuucichthys* gen. nov.

#### 3.1.1. Diagnosis

A soft-bodied vertebrate exhibiting the following unique combination of characters: torpedo-shaped body approximately four times longer than deep with a mid-length ventral notch; anterior head region well differentiated, elongated and bearing a pair of large, anterolaterally projecting eyes; large branchial cavity, occupying the anteroventral quarter of the body in lateral view, bordered ventrally by a keel, and possibly associated dorsally with up to seven branchial bars; approximately 25 straight to gently posteriorly curved myomeres, including particularly thick anterior ones; elongated liver-like internal organ; short spiniform caudal process, but no fins.

#### 3.1.2. Type species

*Nuucichthys rhynchocephalus* gen. et sp. nov. (by monotypy).

#### 3.1.3. Etymology

Concatenation of *Núu-ci*, the name Utes give to themselves (the *Núu-ci* meaning ‘The People’) [36], and *ichthys*, meaning ‘fish’ in Greek, in reference to the discovery of the holotype on what is historically Pahvant ‘Ute’ territory.

#### 3.1.4. Remarks

**Figure 1.**
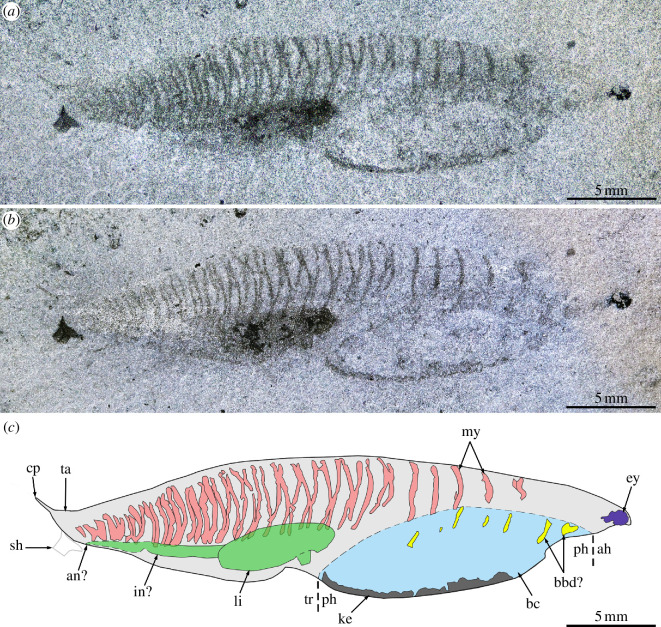
*Nuucichthys rhynchocephalus* gen. et sp. nov. from the Drumian Marjum Formation of the House Range of Utah, USA. (*a,b*) General views of the holotype (UMNH.IP.6084), which was photographed dry with direct light (*a*) or immersed in dilute ethanol with cross-polarized light (*b*). (*c*) Interpretative drawing combining details of (*a,b*). Abbreviations: *ah*, anterior head region; *an*, anus; *bbd*, dorsal branchial bar; *bc*, branchial chamber; *cp*, spiniform caudal process; *ey*, eye; *in*, intestine; *ke*, keel; *li*, liver; *my*, myomere; *ph*, posterior head region; *sh*, shelly fragment; *ta*, tail; *tr*, trunk region.

**Figure 2.**
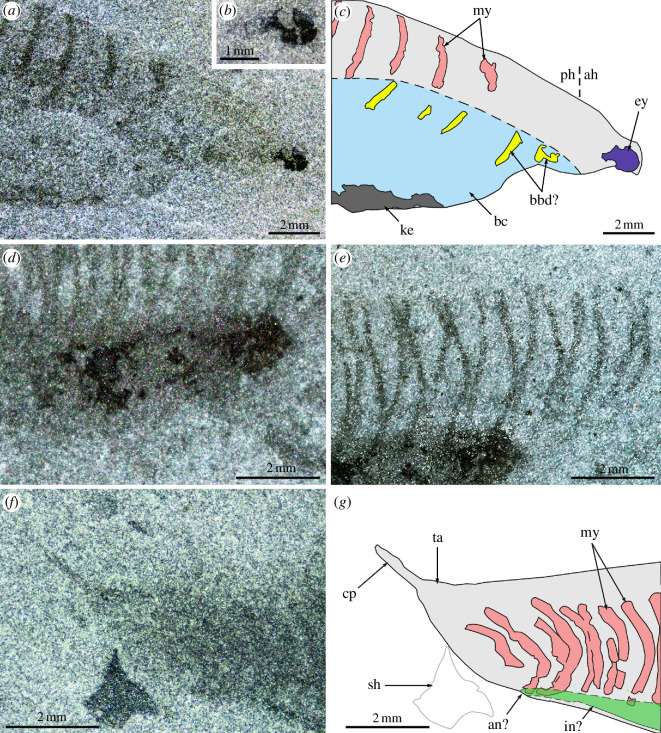
*Nuucichthys rhynchocephalus* gen. et sp. nov. from the Drumian Marjum Formation of the House Range of Utah, USA. (*a*,*b*,*d–f*) Detailed views of the anterior region of the body (*a*), eye (*b*), possible liver (*d*), myomeres (*e*), and posterior trunk region (*f*) in specimen UMNH.IP.6084; specimen dry (*a,b,f*) or immersed in dilute ethanol (*d,e*) illuminated with direct light. (*c,g*) Interpretative drawings of (*a,f*). Abbreviations: *ah*, anterior head region; *an*, anus; *bbd*, dorsal branchial bar; *bc*, branchial chamber; *cp*, spiniform caudal process; *ey*, eye; *in*, intestine; *ke*, keel; *my*, myomere; *ph*, posterior head region; *sh*, shelly fragment; *ta*, tail.

*Nuucichthys* gen. nov. is most similar to *Metaspriggina* [37] from the Miaolingian of British Columbia in the general shape and proportions of the body in lateral view (e.g. [4, fig. 1*h*,*i*,*k*, extended data figs 2*e* and 3]). However, the anterior (pre-branchial) head region of *Nuucichthys* is elongated and well differentiated from the posterior (branchial) head region. By contrast, the distance between the eyes and the anteriormost branchial bar indicates that the anterior head region is short in *Metaspriggina*, which along with the presence of myomeres in this area makes it hardly distinguishable from the rest of the body (e.g. [4, figs 1*a–c*,*d* and 2, extended data fig. 5*c*]). The eyes of *Nuucichthys* project anterolaterally, whereas they are reconstructed as dorsal in *Metaspriggina* [4] (but see §4.2.2). *Nuucichthys* has noticeably fewer (approx. 25) and anteriorly thicker myomeres than *Metaspriggina* (approx. 40), and a spiniform caudal termination that remains unreported in the latter Canadian taxon.

*Nuucichthys* is also comparable to *Emmonsaspis* [5] from the lower Cambrian Parker Slate Formation and possibly the Kinzers Formation [4, extended data fig. 6, their ‘*Metaspriggina* spp.’]. However, *Emmonsaspis* has a deeper body and more numerous (>50) tightly packed and chevron-shaped myomeres compared with *Nuucichthys*, and a barely differentiated anterior head region akin to *Metaspriggina*.

*Nuucichthys* and the lower Cambrian *Myllokunmingia* [34] (including its possible junior synonym *Haikouichthys*) share the apparent absence of myomeres in the anterior head region [31] but are otherwise easy to distinguish. *Nuucichthys* differs from *Myllokunmingia* by its larger eyes, its much thicker myomeres and its lack of dorsal and ventral fins.

Finally, *Nuucichthys* is easily distinguished from *Yunnanozoon* [38] by its well-differentiated anterior head region, and its lack of filament-bearing branchial arches and ‘dorsal repetitive units’, two particularly prominent characteristics of the Chengjiang taxon recently re-interpreted as a stem-group vertebrate [3] (but see [24]).

*Nuucichthys rhynchocephalus* sp. nov.

#### 3.1.5. Diagnosis

As for the genus.

#### 3.1.6. Etymology

From the Greek *rhynchos* and *kephale*, meaning ‘snout’ or ‘beak’ and ‘head’, respectively, in reference to the protruding anterior head region.

#### 3.1.7. Material, locality and horizon

The holotype and only specimen (UMNH.IP.6084), a complete, laterally flattened individual (part only); light-grey shale of the Cambrian (Miaolingian: Drumian) Marjum Formation, most probably its middle part (*Ptychagnostus punctuosus* agnostoid biozone); indeterminate locality in the House Range of Utah, USA.

#### 3.1.8. Description

**Figure 3.**
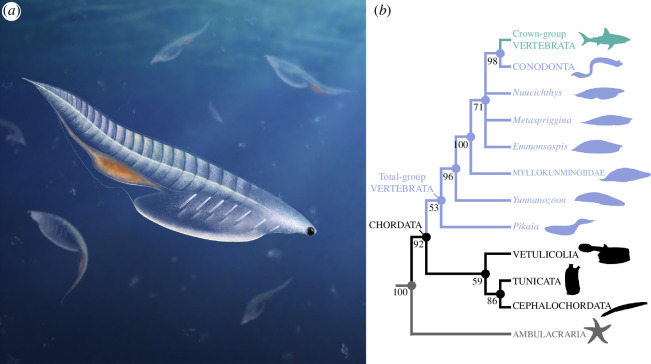
*Nuucichthys rhynchocephalus* gen. et sp. nov. and its phylogenetic position within deuterostomes. (*a*) Reconstruction of the animal in its putative living environment (credit: Franz Anthony). (*b*) Simplified topology recovered from the analysis of a modified version of Tian *et al*.’s dataset [3] through Bayesian inference (see electronic supplementary material, figure S1, for complete tree). Numbers at the nodes indicate posterior probability values. Note that the phylogenetic placement of vetulicolians remains highly debatable, but the exclusion of these organisms from our analysis resulted in essentially the same tree topology (electronic supplementary material, figure S2).

The main body is fusiform, 32.4 mm in length (excluding caudal process and eye) and 7.9 mm in maximum height (at about mid-length) (figure 1*a–c*). The anterior head region is elongated (approx. 30% longer than wide) and bears a large eye bulging out anterolaterally (figure 2*a*−*c*). The rest of the body features a branchial chamber and a prominent internal organ ventrally, and a series of approximately 40 thin roughly transverse bands dorsally, interpreted as myomeres that have shrunk after death. The branchial chamber is large (approx. 39% and 67% of the maximum length and height of the main body, respectively) and noticeably protrudes ventrally, thus forming re-entrants with the ventral margins of both the anterior head region and the trunk region; this chamber is bounded ventrally by an unevenly thick keel (figure 1*a–c*). Faintly expressed, small elongated elements located in the dorsal region of the branchial chamber are tentatively interpreted as dorsal branchial bars [4] (figures 1*a–c* and 2*a,c*). Possibly seven bars are present, including five posterior ones located close, but noticeably offset to the five anteriormost shrunken myomeres, and two anterior bars positioned farther anteriorly than any myomeres. The large internal organ, expressed as an elongated ellipsoidal dark structure located immediately posterior to the branchial cavity (figures 1*a–c* and 2*d*), is tentatively interpreted as the liver, following Conway Morris & Caron [4]. It is orientated with its long axis forming an angle of 15° with the anteroposterior axis of the body. Posterior to this organ, a faint narrow stripe is visible, possibly the intestine, which seems to meet the ventral body margin (anus?) opposite the posteriormost myomere ventrally (figures 1*a–c* and 2). The five anteriormost shrunken myomeres are short, widely spaced and subparallel (figure 2*a,c*). They are followed by more closely spaced bands forming a zigzag pattern (figure 2*e*), a motif that progressively disappears posteriorly as myomeres shorten and the spacing between them reduces (figure 1*a–c*). The zigzag pattern results from the offsetting of the right and left series of half-myomeres, which is mostly present in the middle section of the trunk; once this offsetting is accounted for, the total number of myomeres is close to 25. The posterior region of the body is filled with small, densely packed myomeres, and terminated by a short spiniform process that projects posterodorsally at a 35° angle relative to the anteroposterior axis of the body (figure 2*f,g*). A morphological reconstruction is presented in figure 3*a*.

### 3.2. Phylogenetic analysis

The Bayesian inference analysis of the modified dataset of Tian *et al*. [3] recovered *Nuucichthys* gen. nov. within the stem lineage of vertebrates, forming a polytomy with *Emmonsaspis*, *Metaspriggina*, and a clade composed of conodonts and crown-group vertebrates (figure 3*b*; electronic supplementary material, figure S1). Other stem-group vertebrates, namely the myllokunmingiids (*Myllokunmingia* and its junior synonym ‘*Haikouichthys*’), *Yunnanozoon* and *Pikaia* occupy increasingly stem-ward positions relative to *Nuucichthys*. The topology resulting from our analysis is congruent with that of Tian *et al*. [3], except for the stem-ward position of myllokunmingiids relative to *Metaspriggina*, and the grouping of vetulicolians and non-vertebrate chordates, rather than a placement of this group as stem-group chordates or in polytomy with chordates and non-chordate deuterostomes (figure 3*b*). These differences do not impact the interpretation of *Nuucichthys* as an early vertebrate. The exclusion of vetulicolians from the dataset does not change the phylogenetic placement for *Nuucichthys*, and results in essentially the same topology for the whole phylogenetic tree, except for a couple of minor changes within crown-group Vertebrata (electronic supplementary material, figure S2).

## 4. Discussion

### 4.1. Impact of decay on the fossilized morphology

The discovery of *Nuucichthys* gen. nov. is a valuable contribution to early vertebrate evolution and biodiversity given the paucity of these organisms in Cambrian sites with exceptional fossil preservation. However, it is necessary to cautiously interpret the fossilized morphology, as early branching vertebrates have been shown to be highly susceptible to taphonomic biases, such as stem-ward slippage, as evidenced by decay patterns in extant representatives [39–41]. The new fossil shows clear evidence of preservation of delicate cellular organs such as the eyes, myomeres and digestive system, that can become either altered or entirely lost during the early stages of decay. A comparison with decay patterns of amphioxus and modern vertebrates allows a tentative assessment of the duration and impact of decay in the Marjum specimen.

#### 4.1.1. Eyes

**Figure 4.**
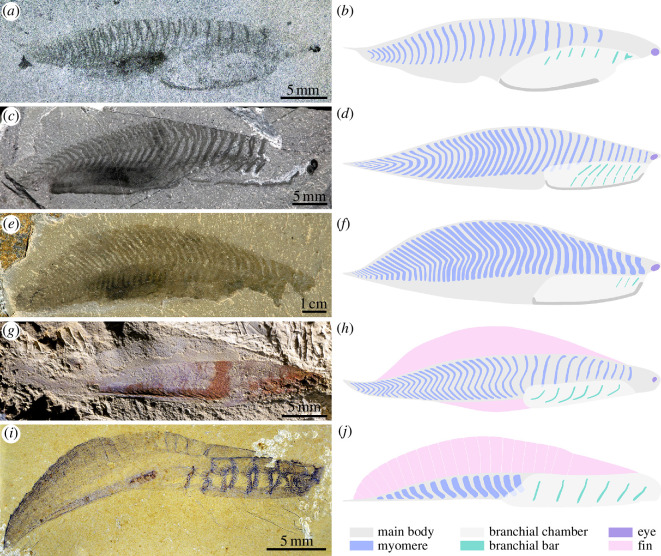
Diversity of Cambrian non-biomineralizing stem-group vertebrates. (*a,b*) *Nuucichthys rhynchocephalus* gen. et sp. nov., Drumian Marjum Fm., Utah, USA. (*a*) Holotype UMNH.IP.6084, general view (dry, direct light). (*b*) Morphological reconstruction; note that the presence of dorsal branchial bars in this taxon is uncertain. (*c,d*) *Metaspriggina walcotti* Simonetta & Ineson [37], Wuliuan Burgess Shale Formation, British Columbia, Canada. (*c*) ROMIP 65347, general view (dry, polarized light); this specimen is incompletely preserved anterodorsally, resulting in the absence of the anteriormost myomeres and branchial bars. (*d*) Morphological reconstruction. (*e,f*) *Emmonsaspis cambrensis* (Walcott) [42], Cambrian Stage 4 Parker Slate Fm., Vermont, USA. (*e*) USNM 15314a (specimen 1), general view (dry, polarized light); this specimen only preserves the trunk. (*f*) Morphological reconstruction. (*g,h*) *Myllokunmingia fengjiaoa* Shu *et al.* [in 31], Cambrian Stage 3 Chiungchussu Fm., Yunnan, China. (*g*) RCCBYU 10200a, general view (dry, low-angle direct light); note that some details of the anterior head are not visible in this specimen. (*h*) Morphological reconstruction. (*i,j*) *Yunnanozoon lividum* Hou *et al.* [43], Cambrian Stage 3 Chiungchussu Formation Fm., Yunnan, China. (*i*) RCCBYU 10310a, general view (dry, direct); note that the caudal process is missing in this specimen. (*j*) Morphological reconstruction; note that the number and distribution of the myomeres are tentative. The reconstructions in (*b,d,f,h,j*) only illustrate some discriminating anatomical features discussed in the text, as they would be preserved in fossils (i.e. including decay changes, such as the shrinkage of myomeres), rather than the living organisms. Images in (*c,e,i*) were mirrored to facilitate comparison with the new fossil, and those in (*g,i*) were first published in [38] and [44], respectively. Credits: J.-B. Caron for (*c,e*) and D. J. Siveter for (*g,i*).

The appearance and size of the eye preserved as a thick carbonaceous film on the side of the head of UMNH.IP.6084 suggests that *Nuucichthys* probably possessed complex eyes with lenses and visual pigments akin to those of lampreys. This interpretation is consistent with the presence of similarly positioned large camera-style eyes in the closely related *Metaspriggina* [4] (figure 4*c,d*). In lampreys, the loss of the eyes starts eight days after death in larvae, which are comparable in size to the Marjum fossil or larger (<8 cm), and 35 days after death in the much larger adults [41]. By comparison, the simpler eye spots of extant amphioxus disappear after two days of decay only [41].

#### 4.1.2. Feeding and branchial apparatus

We find no evidence of feeding structures in UMNH.IP.6084, such as keratinous teeth or cartilages, which would be expected to resist decay for relatively long periods of time considering their robust nature [41]. As such, we consider their absence in *Nuucichthys* as a legitimate biological signal. We suggest the presence of up to seven putative branchial bars in *Nuucichthys* based on the preservation of faint rod-shaped carbonaceous impressions within the pharyngeal region of the holotype. However, these features are comparatively less well preserved relative to other parts of the anatomy (e.g. myomeres, viscera, eyes), which concurs with the experimental work showing that the branchial support system is readily lost during decay (within days) in lampreys and hagfish [41].

#### 4.1.3. Muscles

The presence of numerous myomeres in UMNH.IP.6084 is consistent with the long persistence of muscle blocks in extant cephalochordates (amphioxus) and vertebrates (e.g. lamprey, hagfish) [41]. The variations in spacing and organization of these structures along the anteroposterior axis can be explained by a combination of legitimate anatomical differences and a differential impact of decay. The presence of gaps between the myomeres of UMNH.IP.6084 mirrors the conditions exhibited by most fossils of Cambrian chordates and can be interpreted as evidence of post-mortem shrinkage (e.g. [4,45]). The latter transformation starts within four to eight days of decay in modern taxa, where it is associated with the rapid loss of the more fragile ventral parts of the myomeres and the resulting change from a W-shaped configuration of the myomeres to a Z- or V-shaped one [41]. The greater disorganization of the myomeres in the middle part of the body of UMNH.IP.6084 suggests a more advanced decay in this region (see [41, fig. 3*e*] for comparison). However, the gradual decrease in size of the gaps separating the myomeres posteriorly is suggestive of a legitimate trait of the anatomy of *Nuucichthys*: a decrease in the thickness of the muscle blocks posteriorly in the living organism. Similarly, the gradual decrease in height anteriorly of the shrunken myomeres in the posterior cephalic region suggests that the absence of myomeres in the anterior cephalic region is probably legitimate.

#### 4.1.4. Axial structures

We find no evidence for the preservation of axial structures in *Nuucichthys*, specifically the notochord. This key synapomorphy of chordates is rapidly lost after death in amphioxus, but has proved particularly resistant to decay in the experimental studies of modern vertebrates [39,45]. Yet, its preservation in stem-group vertebrates from Cambrian Konservat-Lagerstätten is exceedingly rare. In the illustrated materials of *Metaspriggina*, a thin continuous longitudinal structure convincingly representing a notochord is visible in one specimen from the Burgess Shale (out of 15) and possibly two specimens from the Duchesnay Unit (out of 44) (e.g. [4, fig. 1*e*]). The remains of notochords identified by Conway Morris and Caron in four additional specimens are hardly distinguishable from the myomeres and would represent particularly incomplete notochords. Likewise, none of the published specimens of *Emmonsaspis* convincingly display remains of notochords [4,8]. Among myllokunmingiids, the notochord has been tentatively identified in *Myllokunmingia* [31], but this interpretation has been repeatedly challenged [34,38]. In conclusion, despite the seemingly high preservation potential of the notochord as informed by empirical work, understanding the controls behind the fossilization of this key feature remains challenging.

#### 4.1.5. Viscera

The holotype of *Nuucichthys* preserves visceral remains that most probably correspond to the gut tract and possibly the liver, based on their sizes, shapes and positions within the body cavity (see [4] for a similar interpretation). Experimental data indicate that the gut tract decays rapidly, particularly when the integrity of its wall has been compromised [41]. According to taphonomy experiments, the liver starts to disappear after eight days in amphioxus, and 15 days in larval and adult lampreys and hagfish [41]. If our interpretation is correct, the relatively good preservation of this organ (well-defined shape, original position) would suggest that the decay of UMNH.IP.6084 did not exceed a couple of weeks.

#### 4.1.6. Fins

We find no traces of dorsal or caudal fins in *Nuucichthys*, despite the colour difference between the fossil and the surrounding matrix along most of the body margins. Experimental taphonomy demonstrates that the fins are moderately resistant to decay in vertebrates, usually more so than other delicate features such as the viscera and eyes [41]. This resistance lasts for a minimum of 8, 35 and 207 days for the caudal fins of hagfish, larval lampreys and adult lampreys, respectively, and a minimum of 15 and 35 days for the dorsal fins of adult lampreys and larval lampreys, respectively [41].

In conclusion, precisely bracketing the duration of decay undergone by UMNH.IP.6084 using the decay patterns of modern cephalochordates and vertebrates is challenging, as these patterns greatly vary depending on the individual, the growth stage and the species concerned. Moreover, the exceedingly rare preservation of the notochord in fossils of stem-group or even crown-group vertebrates indicates that caution must be exercised when interpreting fossilized anatomy in the light of decay experiments only [46]. Considering all these factors, we hypothesize that decay lasted between a few days, as attested by the shrinkage and partial displacement of myomeres, and a couple of weeks, as suggested by the relatively good preservation of labile organs (large eyes and liver). In larval and adult lampreys, this amount of time is potentially enough for the complete decay in most or all specimens of several skeletal (arcualia, branchial cartilage, hyoid) and non-skeletal (e.g. brain, buccal tentacles, heart, pineal organ, sensory lines, velum, shape and symmetry of the gills, their openings or their lamellae) features [41]. In hagfish, it may result in the complete loss of non-skeletal features only (e.g. gill asymmetry and shape, hearts, hypophysis, mouth, post-anal tail, slime glands) [41]. A legitimate absence of all these characters cannot be demonstrated in *Nuucichthys*. Some of these characters are phylogenetically informative (e.g. arcualia) and their presence in better preserved specimens would result in a more crownward phylogenetic placement of the new taxon. Finally, a few days to a couple of weeks is not enough for the complete decay of caudal and/or dorsal fins in most specimens of lampreys and hagfish [41] and, therefore, the absence of fins in *Nuucichthys* appears legitimate.

### 4.2. Morphofunctional and palaeoecological considerations

*Nuucichthys* gen. nov. and its close Cambrian relatives *Emmonsaspis*, *Metaspriggina* and *Myllokunmingia* (including *Haikouichthys*) exhibit a similar general morphology including an elongated lenticular body, paired eyes, myomeres restricted to the dorsal region anteriorly, a well-developed anteroventral branchial chamber and ventral internal organs in the trunk region. These similarities could point to comparable lifestyles in all these Cambrian vertebrates. However, *Nuucichthys* has also unique or rarely observed features that may further inform its ecology and evolutionary significance.

#### 4.2.1. Cephalization

*Nuucichthys* is unique among Cambrian vertebrates in possessing a long, snout-like anterior head region, which is well differentiated from the posterior head region by an abrupt change in body depth and a lack of myomeres (figures 2*a,c* and 4*a,b*), unlike those of *Emmonsaspis* and *Metaspriggina* (figures 2*a,c* and 4*c–f*). When observed laterally, the body of the latter two taxa regularly tapers anteriorly up to the insertion sites of the eyes, which are located only a short distance ahead of the anteriormost myomere and branchial bar (e.g. [4, fig. 1 and extended data fig. 6*c*,*d*]). *Myllokunmingia* is more comparable to *Nuucichthys* in this regard, for the eyes are well separated from the anteriormost branchial element (e.g. [32, fig. 1*e*,*g*,*j*,*l*]), evidencing the development of a distinct anterior head region (figure 4*g,h*). A deflection of the dorsal margin of the body has also been evocated as a marker of the boundary between anterior head region and posterior head region in this taxon (head/trunk boundary in [34]). However, a specimen recently illustrated by Hou *et al*. [38] shows that this deflection does not mark an increase in depth of the body proper, but the transition from the finless anterior body to the dorsal fin (figure 4*g*). As to the myomeres of *Myllokunmingia*, they occur at least as far anteriorly as the limit between posterior head region and anterior head region, but their presence more anteriorly cannot be excluded [34].

#### 4.2.2. Lifestyle and feeding ecology

Our reconstruction of the eyes of *Nuucichthys* projecting anterolaterally from the anterior head region is supported by the cephalic outline in the laterally preserved holotype, which strongly suggests that the dorsal part of the head between the eyes was topographically higher than these organs (figures 2*a–c* and 4*a,b*). In *Metaspriggina*, the eyes appear subdiscoidal in specimens preserved laterally, but elongated elliptical in individuals flattened dorsoventrally (e.g. compare fig. 1*b*,*c* and fig. 1*d* in [4]). This indicates that in this taxon the eyes were only slightly tilted dorsally, their visual surfaces predominantly facing anterolaterally like in *Nuucichthys*. Considering this orientation of the eyes and the laterally compressed body shape [4], the anatomy of *Metaspriggina* does not appear particularly indicative of a strictly eudemersal lifestyle (contra [37, p. 40]), and this might also be true of *Nuucichthys*. In fact, Rival *et al*.’s hydrodynamic analysis of *Metaspriggina* body concluded that it ‘may not necessarily be optimized for cruising near the sea floor’ [47, p. 43].

#### 4.2.3. Locomotion

All Cambrian soft-bodied vertebrates have been reconstructed as active swimmers that propelled their elongated bodies via lateral undulations [4,31,32,38,47,48], but these organisms differ with regard to various anatomical aspects thought to impact swimming capabilities. For instance, cruising aptitude is supported by the descriptions of a large sail-like dorsal structure in *Yunnanozoon* (figure 4*i,j*) (‘dorsal fin’ in [48]), and a dorsal fin and a ventral fin in *Myllokunmingia* (figure 4*g,h*) [31,34], but no fin-like structures were ever observed in *Emmonsaspis* or *Metaspriggina*. This absence of fins was tentatively regarded as taphonomic [4], an explanation that appeared even more likely that none of the specimens illustrated to date preserves a complete body outline. In the holotype of *Nuucichthys*, a colour difference is visible between the marginal areas of the fossil and the surrounding matrix. The outline of the body appears well delimitated ventrally, especially along the branchial chamber where it is highlighted by the keel (figure 1). Posteriorly, the ventral body margin forms a strong re-entrant, then runs near the putative liver and progressively tapers towards the caudal end, nowhere forming a distinct ventral fin. A similar ventral condition is convincingly documented in *Metaspriggina* (figure 4*c,d*) and at least the presence of a keel appears likely in *Emmonsaspis* (figure 4*f*) [4, extended data fig. 6*c*]. The dorsal margin is not clearly expressed near the mid-length region of the body in UMNH.IP.6084, but it can be confidently followed farther anteriorly and posteriorly (figure 1). This dorsal margin runs close to the dorsal extremities of the myomeres, again nowhere forming a distinct extension of the body that could be regarded as a fin. As discussed above, the preservation of the eyes, liver and possible remains of the intestine in UMNH.IP.6084—organs equally or even less resistant to decay than the fins in hagfish and larval and adult lampreys [41]—strongly argues against the absence of fins being a taphonomic artefact. In the light of these different observations, and considering the overall great similarity in body organization of the three Laurentian taxa, the hypothesis of *Emmonsaspis* and *Metaspriggina* being *bona fide* fin-less organisms appears most likely [4,37,47] (*contra* [49]). The absence of fins suggests that the swimming mode of these three Laurentian forms would probably significantly differ from that of the two fin-bearing Chinese taxa.

Another interesting feature of *Nuucichthys* is its short spiniform process at the caudal end of the body (figures 2*f,g* and 4*a,b*). A similar structure (figure 4*j*) has been described in *Yunnanozoon* under various names, such as ‘tail tip’ [50], ‘caudal projection’ [51], or ‘posterior projection’ [52]. The strong tapering of the caudal region in rare specimens of *Metaspriggina* suggests that it might have borne a similar process [4, fig. 1*a*,*b*]. As pointed out by Rival *et al*. [47], caudal morphology can dramatically change the mechanics and hydrodynamics of swimming in modern animals. It seems reasonable to assume that the evolution of caudal processes in some early vertebrates resulted in enhanced swimming efficiency.

In summary, we reconstruct *Nuucichthys* as a soft-bodied stem-group vertebrate with a well-developed elongated anterior head region, large and mostly lateral eyes, no fins but a short caudal process. This suite of characters speaks to a life in the water column with limited swimming abilities or, in other words, a planktonektic lifestyle. Hypothesized for *Metaspriggina* as well [53], this lifestyle would be compatible with microphagous feeding habits, including suspension-feeding.

### 4.3. The fossil record of early vertebrates in Laurentia

**Figure 5.**
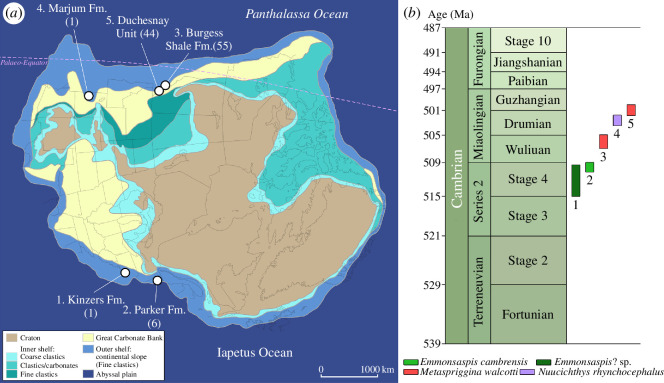
Palaeobiogeography and biostratigraphy of Cambrian soft-bodied vertebrates of Laurentia. (*a*) Distribution of Konservat-Lagerstätten yielding soft-bodied vertebrate fossils in Laurentia; number in parentheses in (*a*) refers to the number of specimens known for each occurrence according to [4,59] and the present study; background map modified from [60]. (*b*) Biostratigraphic distribution of the Cambrian soft-bodied vertebrates of Laurentia; numbers in (*b*) refer to the numbering of the deposits in (*a*); durations of geochronologic units and approximate ages of their boundaries are from [61].

The Cambrian system of Laurentia has yielded three free-living soft-bodied chordates, namely *Emmonsaspis*, *Metaspriggina* and *Pikaia*. Whereas the precise phylogenetic position of *Pikaia* within total-group Chordata remains disputed (e.g. [2–4,24,45,54]), there is a growing consensus that *Metaspriggina* represents a stem-group vertebrate [2,3,55–58] (this study). The anatomy of the rarer *Emmonsaspis* is imperfectly known, but it is in various ways comparable to that of *Metaspriggina* [4,8], which concurs with the recovery of these two taxa as part of a polytomy in our cladistic analysis. The discovery of the closely related *Nuucichthys* in the American Great Basin region complements previous reports of entirely soft-bodied stem-group vertebrates in British Columbia and northeastern USA and in doing so, confirms the wide palaeogeographic distribution of these organisms around the palaeocontinent Laurentia [4] (figure 5*a*). However, their abundance greatly varies from one fossil site to another or even within a given deposit. Thus, most of the *Metaspriggina* specimens (44 out of 55) recovered from the Burgess Shale Formation originate from the Marble Canyon locality. Similarly, 44 *Metaspriggina* specimens have been found in the younger Duchesnay Unit at Haiduk Cirque, but none in the same lithostratigraphic unit at Tangle Peak [4,7]. All these specimens from Haiduk Cirque occur on a single slab, which suggests that environmental stress severe enough to provoke a mass mortality event is responsible for their exceptional preservation. Otherwise, *Emmonsaspis* may be regarded as relatively common at the Parker Slate Quarry (six specimens), considering the limited number of soft-bodied fossils recovered from this site [8]. By contrast, soft-bodied stem-group vertebrates have proved particularly elusive in the Kinzers and Marjum formations, a single specimen being known in each case to this date [4] (this study).

The emerging picture is that of a palaeogeographically wide, but sporadic distribution of early vertebrates within the Cambrian deposits of Laurentia, which could be owing to habitat preferences, a pelagic (planktonektic?) lifestyle, taphonomy or a combination of these factors. These organisms are as yet solely known from quiet, relatively deep-water deposits of the shelf break similar to most Cambrian Burgess Shale-type localities; so if palaeoenvironmental parameters were key to their preservation, they might have been more complex than bathymetry alone (see [62] for a discussion on spatial heterogeneity of benthic communities in the Burgess Shale). A pelagic lifestyle, combined with a body almost exclusively composed of labile tissues and, therefore, particularly prone to post-mortem degradation, could explain why these taxa were fossilized so rarely, even in Konservat-Lagerstätten with broadly similar physical and geochemical characteristics. The earliest vertebrates would have only entered the fossil record under the most exceptional circumstances, such as a great abundance of carcasses (e.g. Haiduk Cirque mass mortality slab) and/or environmental conditions highly conducive to the rapid preservation of carbonaceous remains (e.g. at Marble Canyon [63]). The discovery of *Nuucichthys* in the House Range of Utah confirms that such circumstances occasionally occurred during the deposition of the Marjum Formation, as already suggested by the descriptions of other pelagic organisms with exceedingly scarce fossil records, such as jellyfish, comb jellies and even another chordate [64–66].

*Nuucichthys* also complements the known stratigraphic range of early vertebrates in Laurentia, which is now almost continuous from the Cambrian Stage 4 to the terminal Drumian/basal Guzhangian (figure 5*b*). If Conway Morris & Caron [4, extended data fig. 7] suggested a basal Drumian age for the youngest occurrence of *Metaspriggina* in the Duchesnay Unit, a terminal Drumian (upper *Ptychagnostus punctuosus* agnostoid biozone) to basal Guzhangian (lower *Lejopyge laevigata* agnostoid biozone) age appears more likely, considering the co-occurrence in these strata with the agnostoid *Lejopyge calva* [7,67]. The Duchesnay Unit and the Burgess Shale occurrences of *Metaspriggina* are, therefore, separated by more than three million years, a gap in the Laurentian fossil record of stem-group vertebrates now filled by the discovery of *Nuucichthys* (lower *Ptychagnostus punctuosus* agnostoid biozone; figure 5*b*).

## 5. Conclusions

The Drumian strata of the Marjum Formation keep yielding exceptionally preserved soft-bodied taxa previously unreported in the region, as well as forms entirely new to science (e.g. [13,16,17,68]). The Marjum Konservat-Lagerstätte shares the distinction of being one of a few Cambrian deposits to preserve remains of organisms as delicate as comb jellies [65], jellyfish [64], tunicates [66] and now non-biomineralizing vertebrates (this study) with Chengjiang [38], Qingjiang [69] and the Burgess Shale [4,70]. The Marjum biota differs from the remarkable fossil assemblages of the underlying Wheeler Formation and the overlying Weeks Formation by its overall better preservation, as well as its high proportion of free-swimming components [13], both aspects further evidenced by the discovery of *Nuucichthys*. How these two characteristics relate is unclear but taken together, they suggest that the Marjum Formation may uniquely contribute to a better understanding of how macroscopic animals fundamentally transformed marine pelagic ecosystems in the Cambrian.

### 5.1. Data and software availability

Nomenclatural acts relating to the new taxon are registered on ZooBank: urn:lsid:zoobank.org:pub:836237BD-E321-4EA8-94BA-552901EE513D (publication), urn:lsid:zoobank.org:act:235314F4-7517-4D9A-B39D-E544B4CA7804 (genus), urn:lsid:zoobank.org:act:E7C56534-B172-470A-8DBC-B873B088B8D2 (species).

## Data Availability

The fossil specimen described in this contribution is stored in the collections of Invertebrate Palaeontology of the Natural History Museum of Utah (UMNH.IP.6084), Salt Lake City, USA. The phylogenetic analysis dataset and a complete version of the recovered cladogram are available as electronic supplementary material [71].

## References

[1] Erwin DH, Laflamme M, Tweedt SM, Sperling EA, Pisani D, Peterson KJ. 2011 The Cambrian conundrum: early divergence and later ecological success in the early history of animals. Science **334**, 1091–1097. (10.1126/science.1206375) 22116879

[2] Janvier P. 2015 Facts and fancies about early fossil chordates and vertebrates. Nature **520**, 483–489. (10.1038/nature14437) 25903630

[3] Tian Q, Zhao F, Zeng H, Zhu M, Jiang B. 2022 Ultrastructure reveals ancestral vertebrate pharyngeal skeleton in yunnanozoans. Science **377**, 218–222. (10.1126/science.abm2708) 35857544

[4] Conway Morris S, Caron J-B. 2014 A primitive fish from the Cambrian of North America. Nature **512**, 419–422. (10.1038/nature13414) 24919146

[5] Resser CE, Howell BF. 1938 Lower Cambrian Olenellus zone of the Appalachians. Geol. Soc. Am. Bull. **49**, 195–248. (10.1130/GSAB-49-195)

[6] Conway Morris S. 1993 Ediacaran-like fossils in Cambrian Burgess Shale-type faunas of North America. Palaeontology **36**, 593–635. (10.1111/j.0031-0239.1993.00593.x)

[7] Johnston KJ, Johnston PA, Powell WG. 2009 A new, middle Cambrian, Burgess Shale-type biota, Bolaspidella zone, Chancellor Basin, southeastern British Columbia. Palaeogeogr. Palaeoclimatol. Palaeoecol. **277**, 106–126. (10.1016/j.palaeo.2009.02.015)

[8] Pari G, Briggs DEG, Gaines RR. 2022 The soft-bodied biota of the Cambrian Series 2 Parker Quarry Lagerstätte of northwestern Vermont, USA. J. Paleontol. **96**, 770–790. (10.1017/jpa.2021.125)

[9] Gaines RR. 2014 Burgess Shale-type preservation and its distribution in space and time. Paleontol. Soc. Pap. **20**, 123–146. (10.1017/S1089332600002837)

[10] Lieberman BS. 2003 A new soft-bodied fauna: the Pioche Formation of Nevada. J. Paleontol. **77**, 674–690. (10.1666/0022-3360(2003)077<0674:ANSFTP>2.0.CO;2)

[11] Kimmig J, Strotz LC, Kimmig SR, Egenhoff SO, Lieberman BS. 2019 The Spence Shale Lagerstätte: an important window into Cambrian biodiversity. J. Geol. Soc. Lond. **176**, 609–619. (10.1144/jgs2018-195)

[12] Robison RA, Babcock LE, Gunther VG. 2015 Exceptional Cambrian fossils from Utah: a window into the age of trilobites. Utah Geol. Surv. Misc. Publ. **15**, 1–97.

[13] Pates S, Lerosey-Aubril R, Daley AC, Kier C, Bonino E, Ortega-Hernández J. 2021 The diverse radiodont fauna from the Marjum Formation of Utah, USA (Cambrian: Drumian). PeerJ **9**, e10509. (10.7717/peerj.10509) 33552709 PMC7821760

[14] Lerosey-Aubril R, Gaines RR, Hegna TA, Ortega-Hernández J, Van Roy P, Kier C, Bonino E. 2018 The Weeks Formation Konservat-Lagerstätte and the evolutionary transition of Cambrian marine life. J. Geol. Soc. Lond. **175**, 705–715. (10.1144/jgs2018-042)

[15] Lerosey‐Aubril R, Kimmig J, Pates S, Skabelund J, Weug A, Ortega‐Hernández J. 2020 New exceptionally-preserved panarthropods from the Drumian Wheeler Konservat-Lagerstätte of the House Range of Utah. Pap. Palaeontol. **6**, 501–531. (10.1002/spp2.1307)

[16] Leibach WW, Lerosey-Aubril R, Whitaker AF, Schiffbauer JD, Kimmig J. 2021 First palaeoscolecid from the Cambrian (Miaolingian, Drumian) Marjum Formation of western Utah. Acta Palaeontol. Pol. **66**, 663–678. (10.4202/app.00875.2021)

[17] Lerosey‐Aubril R, Maletz J, Coleman R, Del Mouro L, Gaines RR, Skabelund J, Ortega‐Hernández J. 2024 Benthic pterobranchs from the Cambrian (Drumian) Marjum Konservat-Lagerstätte of Utah. Pap. Palaeontol. **10**, e1555. (10.1002/spp2.1555)

[18] Rees MN. 1986 A fault-controlled trough through a carbonate platform: the middle Cambrian House Range Embayment. Geol. Soc. Am. Bull. **97**, 1054–1069. (10.1130/0016-7606(1986)97<1054:AFTTAC>2.0.CO;2)

[19] Miller JF, Evans KR, Dattilo BF. 2012 The great American carbonate bank in the Miogeocline of western central Utah: tectonic influences on sedimentation. In The great American carbonate bank: the geology and economic resources of the Cambro-Ordovician Sauk sequence of Laurentia, vol. 98 (eds JR Derby, R Fritz, SA Longacre, M Morgan, C Sternbach), pp. 769–854. Tulsa, OK: American Association of Petroleum Geologists. (10.1306/13331516M983498)

[20] Foster JR, Gaines RR. 2016 Taphonomy and paleoecology of the ‘middle’ Cambrian (Series 3) formations in Utah’s West Desert: recent finds and new data. In Resources and geology of Utah’s West Desert, vol. 45 (eds JB Comer, PC Inkenbrandt, KA Krahulec, ML Pinnell), pp. 291–336. Salt Lake City, UT: Utah Geological Association.

[21] Lewis PO. 2001 Phylogenetic systematics turns over a new leaf. Trends Ecol. Evol. **16**, 30–37. (10.1016/S0169-5347(00)02025-5) 11146142

[22] Ronquist F *et al*. 2021 Mrbayes 3.2: efficient Bayesian phylogenetic inference and model choice across a large model space. Syst. Biol. **61**, 539–542. (10.1093/sysbio/sys029) PMC3329765 22357727

[23] Rambaut A, Drummond AJ, Xie D, Baele G, Suchard MA. 2017 Tracer v1.6. See http://beast.community/tracer. 10.1093/sysbio/syy032 PMC6101584 29718447

[24] Mussini G, Smith MP, Vinther J, Rahman IA, Murdock DJE, Harper DAT, Dunn FS. 2024 A new interpretation of Pikaia reveals the origins of the chordate body plan. Curr. Biol. **34**, S0960-9822(24)00669-9. (10.1016/j.cub.2024.05.026) 38866005

[25] Ou Q, Conway Morris S, Han J, Zhang Z, Liu J, Chen A, Zhang X, Shu D. 2012 Evidence for gill slits and a pharynx in Cambrian vetulicolians: implications for the early evolution of deuterostomes. BMC Biol. **10**, 81. (10.1186/1741-7007-10-81) 23031545 PMC3517509

[26] García-Bellido DC, Lee MSY, Edgecombe GD, Jago JB, Gehling JG, Paterson JR. 2014 A new vetulicolian from Australia and its bearing on the chordate affinities of an enigmatic Cambrian group. BMC Evol. Biol. **14**, 214. (10.1186/s12862-014-0214-z) 25273382 PMC4203957

[27] Briggs DEG, Lieberman BS, Halgedahl SL, Jarrard RD. 2005 A new metazoan from the middle Cambrian of Utah and the nature of the Vetulicolia. Palaeontology **48**, 681–686. (10.1111/j.1475-4983.2005.00489.x)

[28] Aldridge RJ, Hou X-G, Siveter DJ, Siveter DJ, Gabbott SE. 2007 The systematics and phylogenetic relationships of vetulicolians. Palaeontology **50**, 131–168. (10.1111/j.1475-4983.2006.00606.x)

[29] Kapli P, Natsidis P, Leite DJ, Fursman M, Jeffrie N, Rahman IA, Philippe H, Copley RR, Telford MJ. 2021 Lack of support for Deuterostomia prompts reinterpretation of the first Bilateria. Sci. Adv. **7**, eabe2741. (10.1126/sciadv.abe2741) 33741592 PMC7978419

[30] Yang Y, Su BA, Ou Q, Cheng M, Han J, Shu D. 2023 An enigmatic structure in the tail of vetulicolians from the Cambrian Chengjiang Biota, South China. Pap. Palaeontol. **9**, e1537. (10.1002/spp2.1537)

[31] Shu DG *et al*. 1999 Lower Cambrian vertebrates from South China. Nature **402**, 42–46. (10.1038/46965)

[32] Shu DG *et al*. 2003 Head and backbone of the early Cambrian vertebrate Haikouichthys. Nature **421**, 526–529. (10.1038/nature01264) 12556891

[33] Holland ND, Chen J. 2001 Origin and early evolution of the vertebrates: new insights from advances in molecular biology, anatomy, and palaeontology. Bioessays **23**, 142–151. (10.1002/1521-1878(200102)23:2<142::AID-BIES1021>3.0.CO;2-5) 11169587

[34] Hou XG, Aldridge RJ, Siveter DJ, Siveter DJ, Feng XH. 2002 New evidence on the anatomy and phylogeny of the earliest vertebrates. Proc. R. Soc. Lond. B **269**, 1865–1869. (10.1098/rspb.2002.2104) PMC1691108 12350247

[35] Haeckel E. 1874 Anthropogenie oder Entwicklungsgeschichte des Menschen. Leipzig, Germany: Engelmann.

[36] Jones SG. 2019 Being and becoming Ute—the story of an American Indian people. Salt Lake City, UT: University of Utah Press. (10.1353/book71628)

[37] Simonetta AM, Insom E. 1993 New animals from the Burgess Shale (Middle Cambrian) and their possible significance for the understanding of the Bilateria. Boll. di Zool. **60**, 97–107. (10.1080/11250009309355797)

[38] Hou X, Siveter DJ, Siveter DJ, Aldridge RJ, Cong P, Gabbott SE, Ma X, Purnell MA, Williams M. 2017 The Cambrian fossils of Chengjiang, China: the flowering of early animal life, 2nd edn. Chichester, UK: Wiley. (10.1002/9781118896372)

[39] Sansom RS, Gabbott SE, Purnell MA. 2010 Non-random decay of chordate characters causes bias in fossil interpretation. Nature **463**, 797–800. (10.1038/nature08745) 20118914

[40] Sansom RS, Gabbott SE, Purnell MA. 2011 Decay of vertebrate characters in hagfish and lamprey (Cyclostomata) and the implications for the vertebrate fossil record. Proc. R. Soc. B **278**, 1150–1157. (10.1098/rspb.2010.1641) PMC3049070 20947532

[41] Sansom RS, Gabbott SE, Purnell MA. 2013 Atlas of vertebrate decay: a visual and taphonomic guide to fossil interpretation. Palaeontology **56**, 457–474. (10.1111/pala.12037)

[42] Walcott CD. 1890 The fauna of the Lower Cambrian or *Olenellus* zone. In Tenth annual report of the director, 1888–1889, pp. 509–774. Washington, DC: United States Geological Survey.

[43] Hou XG, Ramsköld L, Bergström J. 1991 Composition and preservation of the Chengjiang fauna—a Lower Cambrian soft-bodied biota. Zool. Scr. **20**, 395–411. (10.1111/j.1463-6409.1991.tb00303.x)

[44] Hou XG, Aldridge RJ, Bergström J, Siveter DJ, Siveter DJ, Feng XH. 2004 The Cambrian fossils of Chengjiang, China: the flowering of early animal life, 1st edn. Oxford, UK: Blackwell.

[45] Conway Morris S, Caron J-B. 2012 Pikaia gracilens Walcott, a stemgroup chordate from the middle Cambrian of British Columbia. Biol. Rev. **87**, 480–512. (10.1111/j.1469-185X.2012.00220.x) 22385518

[46] Parry LA *et al*. 2018 Soft‐bodied fossils are not simply rotten carcasses—toward a holistic understanding of exceptional fossil preservation. Bioessays **40**, 1700167. (10.1002/bies.201700167) 29193177

[47] Rival DE, Yang W, Caron J-B. 2021 Fish without tail fins—exploring the function of tail morphology of the first vertebrates. Integr. Comp. Biol. **61**, 37–49. (10.1093/icb/icab004) 33690846

[48] Shu DG, Zhang XL, Chen L. 1996 Reinterpretation of Yunnanozoon as the earliest known hemichordate. Nature **380**, 428–430. (10.1038/380428a0)

[49] Conway Morris S. 2008 Redescription of a rare chordate, Metaspriggina walcotti Simonetta and Insom, from the Burgess Shale (Middle Cambrian). J. Paleontol. **82**, 424–430. (10.1666/06-130.1)

[50] Chen JY, Dzik J, Edgecombe GD, Ramsköld L, Zhou GQ. 1995 A possible Early Cambrian chordate. Nature **377**, 720–722. (10.1038/377720a0)

[51] Chen JY, Huang DY, Li CW. 1999 An Early Cambrian craniate-like chordate. Nature **402**, 518–522. (10.1038/990080)

[52] Cong PY, Hou XG, Aldridge RJ, Purnell MA, Li YZ. 2015 New data on the palaeobiology of the enigmatic yunnanozoans from the Chengjiang Biota, Lower Cambrian, China. Palaeontology **58**, 45–70. (10.1111/pala.12117)

[53] Whalen CD, Briggs DEG. 2018 The Palaeozoic colonization of the water column and the rise of global nekton. Proc. R. Soc. B **285**, 20180883. (10.1098/rspb.2018.0883) PMC6083262 30051837

[54] Mallatt J, Holland N. 2013 Pikaia gracilens Walcott: stem chordate, or already specialized in the Cambrian. J. Exp. Zool. B Mol. Dev. Evol. **320**, 247–271. (10.1002/jez.b.22500) 23606659

[55] Suzuki DG, Grillner S. 2018 The stepwise development of the lamprey visual system and its evolutionary implications. Biol. Rev. Camb. Philos. Soc. **93**, 1461–1477. (10.1111/brv.12403) 29488315

[56] Johanson Z, Boisvert CA, Trinajstic K. 2019 Early vertebrates and the emergence of jaws. In Heads, jaws, and muscles: anatomical, functional, and developmental diversity in chordate evolution (eds JM Ziermann, RE Diaz Jr, R Diogo), pp. 23–44. Cham, Switzerland: Springer Nature. (10.1007/978-3-319-93560-7)

[57] Miyashita T, Green SA, Bronner ME. 2019 Comparative development of cyclostomes. In Evolution and development of fishes (eds Z Johanson, C Underwood, M Richter), pp. 30–58. New York, NY: Cambridge University Press. (10.1017/9781316832172)

[58] Miyashita T. 2022 ‘Arch’-etyping vertebrates. Science **377**, 154–155. (10.1126/science.adc9198) 35857554

[59] Pari G, Briggs DEG, Gaines RR. 2021 The Parker Quarry Lagerstätte of Vermont—the first reported Burgess Shale-type fauna rediscovered. Geology **49**, 693–697. (10.1130/G48422.1)

[60] Miall AD, Blakey RC. 2019 The Phanerozoic tectonic and sedimentary evolution of North America. In The sedimentary basins of the United States and Canada, 2nd edn (ed. AD Miall), pp. 1–38. Amsterdam, The Netehrlands: Elsevier.

[61] Peng SC, Babcock LE, Ahlberg P. 2020 The Cambrian period. In Geologic time scale (eds FM Gradstein, JG Ogg, MD Schmitz, GM Ogg), pp. 565–629. Amsterdam, The Netherlands: Elsevier. (10.1016/B978-0-12-824360-2.00019-X)

[62] Nanglu K, Caron J-B, Gaines RR. 2020 The Burgess Shale paleocommunity with new insights from Marble Canyon, British Columbia. Paleobiology **46**, 58–81. (10.1017/pab.2019.42)

[63] Caron J-B, Gaines RR, Aria C, Mángano MG, Streng M. 2014 A new phyllopod bed-like assemblage from the Burgess Shale of the Canadian Rockies. Nat. Commun. **5**, 3210. (10.1038/ncomms4210) 24513643

[64] Cartwright P, Halgedahl SL, Hendricks JR, Jarrard RD, Marques AC, Collins AG, Lieberman BS. 2007 Exceptionally preserved jellyfishes from the middle Cambrian. PLoS One **2**, e1121. (10.1371/journal.pone.0001121) 17971881 PMC2040521

[65] Parry LA, Lerosey-Aubril R, Weaver JC, Ortega-Hernández J. 2021 Cambrian comb jellies from Utah illuminate the early evolution of nervous and sensory systems in ctenophores. iScience **24**, 102943. (10.1016/j.isci.2021.102943) 34522849 PMC8426560

[66] Nanglu K, Lerosey-Aubril R, Weaver JC, Ortega-Hernández J. 2023 A mid-Cambrian tunicate and the deep origin of the ascidiacean body plan. Nat. Commun. **14**, 3832. (10.1038/s41467-023-39012-4) 37414759 PMC10325964

[67] Peng SC *et al*. 2009 The global boundary stratotype section and point (GSSP) of the Guzhangian stage (Cambrian) in the Wuling Mountains, northwestern Hunan, China. Episodes **32**, 41–55. (10.18814/epiiugs/2009/v32i1/006)

[68] Lerosey‐Aubril R, Ortega‐Hernández J. 2022 A new lobopodian from the middle Cambrian of Utah: did swimming body flaps convergently evolve in stem-group arthropods? Pap. Palaeontol. **8**, e1450. (10.1002/spp2.1450)

[69] Fu D *et al*. 2019 The Qingjiang Biota—a Burgess Shale-type fossil Lagerstätte from the early Cambrian of South China. Science **363**, 1338–1342. (10.1126/science.aau8800) 30898931

[70] Conway Morris S, Collins D. 1996 Middle Cambrian ctenophores from the Stephen Formation, British Columbia, Canada. Phil. Trans. R. Soc. Lond. B **351**, 279–308. (10.1098/rstb.1996.0024)

[71] Lerosey-Aubril R, Ortega-Hernández J. 2024 Data from: A long-headed cambrian soft-bodied vertebrate from the american great basin region. Figshare. (10.6084/m9.figshare.c.7358207) PMC11267725 39050723

